# Analyzing the Association between Student Weight Status and School Meal Participation: Evidence from the School Nutrition and Meal Cost Study

**DOI:** 10.3390/nu13010017

**Published:** 2020-12-23

**Authors:** Sarah Bardin, Alice Ann Gola

**Affiliations:** 1 Mathematica, 955 Massachusetts Avenue, Suite 801, Cambridge, MA 02139, USA; 2 US Department of Agriculture, Food and Nutrition Service (FNS), 1320 Braddock Place, Alexandria, VA 22314, USA; aliceann.gola@usda.gov

**Keywords:** body mass index, School Nutrition Meal Cost Study, school meal participation, National School Lunch Program, weight status

## Abstract

Childhood obesity remains a pressing public health concern. Children consume a substantial amount of their caloric intake while in school, making the passage of the Healthy Hunger-Free Kids Act (HHFKA) in 2010 and the subsequent improvements to the school meal standards a key policy change. Using data from the School Nutrition and Meal Cost Study, this paper seeks to re-examine the association between students’ (*N* = 1963) weight status and participation in the National School Lunch Program (NSLP) and School Breakfast Program (SBP) since the implementation of these policy changes to determine whether, and how, this relationship has changed. After controlling for a wide array of student characteristics and school-level fixed effects, findings from the multivariate regression analyses indicate that usual participation in the school meal programs has no clear association with students’ weight status, which contradicts findings from earlier studies conducted prior to the passage of the HHFKA. These findings are discussed in relation to changes in the demographic composition of usual NSLP participants over time.

## 1. Introduction

Obesity rates for children ages 2 to 10 continue to climb. Between 2015 and 2016, approximately 19% of children were affected by obesity [1], and there was a higher prevalence of obesity among school-age children (6 to 19 years old) compared to preschool-age children. Specifically, about 18% of children ages 6 to 11 and 21% of children ages 12 to 19 were classified as obese, compared to 14% of children ages 2 to 5. Recent research demonstrates that BMI in early childhood predicts preclinical cardiometabolic phenotypes (e.g., higher metabolic syndrome risk score, higher arterial stiffness, and increased carotid intima-media thickness) by the time a child is 11 to 12 years old [2]. Another study found that among a sample of 51,505 children, 53% of children with obesity as adolescents were affected by overweight or obesity at age 5 [3]. Given these concerning trends, preventing the onset of obesity in childhood may be critical in preventing later cardiovascular disease [4].

The National School Lunch Program (NSLP) and School Breakfast Program (SBP) provide meals to children during the school day and serve as an important safety net for children from low-income households who are eligible to receive meals at free or reduced-price rates. During a typical school year, children consume a substantial portion of their caloric intake while in school, making the program a potentially important factor in children’s overall health [5]. Indeed, a previous study by Gleason and colleagues found that SBP participation in school year 2004–2005, but not NSLP participation, was related to significantly lower body mass index (BMI) in children in grades 1 through 12. However, subsequent sensitivity analyses showed this finding was driven by non-Hispanic, white children [6].

In school year 2012–2013, updated nutrition standards were implemented for the school meals as part of the Healthy Hunger-Free Kids Act (HHFKA). These standards better aligned the school meal pattern to the Dietary Guidelines for Americans (DGAs) and were designed to increase the nutritional quality of school meals. In addition, these standards established calorie, sodium, and saturated fat limits on the meals offered to students. As a result of this key policy change, school meals have significantly increased in their nutritional quality. That is, school lunches and breakfasts were significantly more nutritious in school year 2014–2015 compared to 2009–2010, as measured by the Healthy Eating Index (HEI) [7,8]. Importantly, the lunches that NSLP participants *consumed* were significantly more nutritious than the lunches that nonparticipants consumed from home or somewhere else [5].

In addition to these improvements in the nutritional quality of school meals, there have been other notable changes in the demographics of school meal participants, since Gleason and Dodd’s previous study was conducted. Since the 2007–2008 school year, participation in the NSLP has declined among students who do not qualify for free or reduced-price meals—a trend that predates the passage of the HHFKA [9]. Compared to students in school year 2004–2005, there was a 23 percentage-point decline in the proportion of paid NSLP participants (students from households with incomes above 185% of poverty), who reported usually eating a school lunch at least 3 times per week in 2014–2015, and a 19 percentage-point decline in the proportion of non-Hispanic, white students over the same time period [5]. These changes in the composition of NSLP participants are important, because a recent study found mixed results when examining the relationship between BMI and school meal participation among children who qualify for free or reduced-price meals (students from households with incomes at or below 185% of poverty) [10].

Given that school meals have become more nutritious since the implementation of the updated meal standards and the composition of students who participate in the meal programs has significantly changed over time, it is worth re-examining the association between children’s BMI and participation in the NSLP and the SBP, as it is likely that these associations have changed since Gleason and Dodd’s original analysis. Specifically, this paper seeks to update the findings from that previous study using data from the School Nutrition and Meal Cost Study (SNMCS) to explore the relationship between student weight status and participation in the NSLP and the SBP after the implementation of the HHFKA. 

## 2. Materials and Methods 

### 2.1. Study Design and Data Sources

The SNMCS included a nationally representative sample of students ages 6 to 19 who attended a public, non-charter school participating in the NSLP during the 2014–2015 school year. As part of the SNMCS, information on student demographics, activity levels, usual eating habits, and household characteristics were collected via student and parent interviews for a sample of 2165 students across 293 schools. All interviews were conducted in person as a computer-assisted personal interview during the school day. Additionally, trained interviewers obtained height and weight measurements for each student, which allowed for the calculation of students’ BMI. All data collection instruments for SNMCS were approved by the Office of Management and Budget and the New England Institutional Review Board. The study also complied with institutional review processes as required by certain school districts. Additional information regarding the study design and collection of these data is available elsewhere [5,11].

### 2.2. Variable Selection

#### 2.2.1. Key Outcomes

All outcomes for this analysis were derived from students’ BMI. In order to obtain accurate height and weight measurements, which were necessary for the BMI calculations, trained interviewers used a portable stadiometer to measure students’ standing height to the nearest centimeter and an electronic digital-display floor scale to measure students’ weight to the nearest 0.2 pounds. To ensure these measurements were precise, a minimum of two weight and height measurements were obtained. If the first two measurements differed substantially, a third measurement was taken.

Among the 2165 students sampled, 1963 had valid height and weight measurements. Of the students with invalid measurements, 80 had missing or questionable information on height, weight, age, or gender, and 122 students had implausible height or weight values, as defined by the World Health Organization’s standards for biologically implausible height and weight values [12]. For the purposes of this analysis, only students with valid height and weight measurements were included (*N* = 1963).

Three outcome variables related to weight status were analyzed: (1) a continuous BMI measure, (2) an indicator for overweight or obese, and (3) an indicator for obesity. The first outcome was a continuous measure of the student’s BMI, which was calculated by dividing students’ weight in kilograms by their height in meters squared. The second and third outcomes were binary indicators for overweight or obese and obesity, respectively. Both binary outcomes were defined based on the BMI-for-age percentile for each student, which was calculated by comparing the student’s BMI with the Centers for Disease Control and Prevention (CDC) age- and sex-specific growth charts. Consistent with CDC standards, students whose BMI-for-age percentile was in the 85th percentile or higher were classified as having overweight or obesity. Students whose BMI-for-age percentile was in the 95th percentile or higher were classified as having obesity [13]. In addition to these three outcomes, models using a BMI z-score were also explored, and findings were consistent with those from the non-standardized BMI outcome. As a result, we have not included the results from the BMI z-score analyses in this paper.

#### 2.2.2. Key Independent Variables

Because BMI is a long-term health outcome, to understand the relationship between participation in the school meals program and students’ BMI, measures of students’ usual participation in the SBP and the NSLP were used. Both students and parents provided information related to a student’s participation in each program via the student and parent survey instruments. Specifically, students in middle and high school were asked three questions: (1) whether they have ever eaten a school lunch, (2) if they usually eat a school lunch at least 3 times per week, and (3) how many days per week they usually eat a school lunch (0–5 days). Parents were also asked the number of days per week (0–5 days) that their child usually eats a school lunch. Based on this information, a continuous usual NSLP participation variable was constructed. Specifically, if a student reported never eating a school lunch (*N* = 411), it was assumed that they usually participated in the NSLP 0 days per week. Otherwise, the student-reported number of days that they usually eat a school lunch was used. In the absence of student-reported information, the parent-reported number of days of usual participation was used. Missing information was imputed for 19 students depending on whether they reported usually eating a school lunch 3 times per week. In particular, when students reported having eaten a school lunch but not usually eating a school lunch 3 times per week (*N* = 10), the number of usual days of NSLP participation was assumed to be 1 day per week. Finally, for students who reported having eaten a school lunch and usually participating at least 3 times per week (*N* = 9), the number of usual days of NSLP participation was assumed to be 4 days per week. A similar set of questions were asked regarding SBP participation, and an analogous usual SBP participation variable was constructed. Students who attended a school that did not offer the SBP (*N* = 110) were assumed to usually participate 0 days per week.

To assess the reliability of the reported information, comparisons between the student-reported and parent-reported days of participation were performed. For approximately 60% of students, the student-reported and parent-reported values for NSLP participation were identical. The remaining 40% of students’ data varied, on average, by 1 to 2 days per week. Similar results were found for the SBP usual participation comparisons. In addition to student- and parent-reported usual participation status, the SNMCS also obtained dietary intakes for each student, which were used to determine participation status on the day of data collection [11]. Because we would expect higher proportions of students who report more frequently participating in the NSLP and SBP to have participated in the meal programs on the day in which dietary intake information was collected, we also compared the usual participation with this measure (see Appendix A Table A1 for more information). For both the NSLP and the SBP, there was a strong correlation between the number of usual days of participating and participation on the dietary intake day, which provided further support to the validity of the student- and parent-reported information.

### 2.3. Statistical Methods

Descriptive analyses were conducted to estimate differences in the characteristics of students by NSLP and SBP participation status. Differences between participants and nonparticipants were flagged if statistically significant at an alpha of 0.05. Pearson chi-square tests were used to test for statistical significance of differences between categorical variables, and two-sided t-tests were used to test for statistical significance of differences between continuous variables.

Multivariate regression models with school-level fixed effects were used to estimate the association between school meal participation and student weight status (Equation (1)).

*Y_it_ = X_it_β +* α*_1_P^B^_it_ +* α*_2_P^L^_it_ + γ_2_S2_it_ + γ_3_S3_it_ +* ⋯ *+ γ_n_Sn_it_ + u_it_* (1)

where:

*Y_it_* = BMI outcome for student i in school t;
*X_it_* = characteristics of student i in school t that may influence BMI;
*P^B^_it_* = number of days per week that student i in school t usually participates in the SBP;
*P^L^_it_* = number of days per week that student i in school t usually participates in the NSLP;
*S2_it_*, *S3_it_* … *Sn_it_* = school indicator variables for whether student i attends school t;
*u_it_* = random error term; and
*β*, α*_1_,* α*_2_, γ_2_, γ_3_*… *γ_n_* = coefficients to be estimated.

The model was estimated using linear regression techniques for the continuous BMI outcome, and logistic regression techniques were used for both the overweight or obese and the obesity outcome models. All calculations were performed in Stata 15 [14], and all analyses were weighted to account for the complex sampling design, in which students were clustered within schools and districts. A fixed-effects model was selected in order to eliminate bias due to potential omitted factors that may vary across schools, such as the presence of competitive foods, open campus policies, and nutrition education outreach activities, among other factors. Additionally, since students who attend the same school tend to live in similar neighborhoods [15], the school-level fixed effects may also reduce bias due to unobserved characteristics of students. Although not presented in this paper, alternative model specifications were explored, including explicitly controlling for a set of school and district characteristics, and consistent results were found across specifications. 

In addition to the school-level fixed effects, all models controlled for a rich set of student and household characteristics, including a number of characteristics that potentially relate to students’ decision to usually participate in the meal programs as well as independently affect their BMI (see Appendix A Table A2 for list of characteristics). These factors were selected based on a review of existing literature, which routinely control for a combination of student sociodemographic characteristics, activity levels, and eating habits [6,16,17,18]. Missing values were imputed for a small number of these characteristics, when the proportion of missingness did not exceed 15%. Additional characteristics, such as the number of sports teams a student participated in in the past year, were considered for inclusion but given high rates of missingness (>15%) were not included in the final set of controls. Given the number of covariates, the variance inflation factor (VIF) for each model was used to detect high levels of multicollinearity. None of the variables included in the final model specification exceeded a VIF of 5. 

## 3. Results

### 3.1. School Meal Participation Rates

Most students usually participated in the NSLP 3 or more days per week with just under half (43%) of students participating every day (Table 1). In contrast, usual participation in the SBP was substantially lower than that of the NSLP. Overwhelmingly, students reported not usually eating a school breakfast (61%), with only 13% of students participating every day.

### 3.2. Characteristics of the Participants and Nonparticipants

Across key sociodemographic characteristics, NSLP and SBP usual participants, defined as students who usually participate 3 or more days per week, were statistically significantly different from usual nonparticipants, defined as participating fewer than 3 days per week (Table 2). In particular, nearly half (48%) of usual NSLP participants identified as non-Hispanic, white, compared to approximately two thirds of usual nonparticipants. Usual NSLP participants were also younger, on average, compared to nonparticipants. Statistically significant associations between food security status and usual participation as well as between income-to-poverty ratios and participation status were also observed. Specifically, 77% of usual NSLP participants were classified as food-secure, whereas 92% of usual nonparticipants were. About 43% of usual NSLP participants came from households with incomes exceeding 185% of the poverty threshold compared to 92% of usual nonparticipants. Similar differences were observed at breakfast.

### 3.3. Characteristics of Students with Overweight and Obesity

Approximately, one-third (35%) of students had a BMI-for-age percentile in the 85th percentile or higher of the growth reference, indicating that they were affected by overweight or obesity, (Table 3). Although similar proportions of males and females were identified as having overweight or obesity overall, amongst middle- and high-school-age students, between 35% and 40% of males were found to be affected by overweight or obesity compared to 30% of females. Overall, about 40% of Hispanic and non-Hispanic, black students were identified as having overweight or obesity compared to about 30% of non-Hispanic, white students.

Approximately 25% of students who usually participated in the NSLP 0 days per week were affected by overweight or obesity, whereas roughly 40% of students who usually participated 3 or more days per week were identified as having overweight or obesity. However, no clear pattern was observed among usual SBP participants.

### 3.4. Results from Multivariate Analysis

After controlling for observable characteristics and school fixed effects, no statistically significant associations between usual participation in the NSLP or the SBP and student weight status were observed (Table 4). 

Subsequent sensitivity analyses explored the relationship between the frequency of usual participation compared to usually participating 0 days per week in the NSLP and the SBP (Table 5). These analyses showed statistically significant and positive associations between some levels of participation in the NSLP but not all. Specifically, holding all other variables constant, compared to not participating in the NSLP, usually participating 2 days per week, 4 days per week, and 5 days per week were statistically significant from 0 days per week in both the continuous BMI model and the overweight or obese model at an alpha of 0.05. In particular, students who usually participate 2 days per week in the NSLP are expected to have BMIs which are approximately 2 points higher, on average, than students who usually participate 0 days per week, holding all else constant. In comparison to participating 0 days per week, students who participate 4 days per week are expected to have BMIs that are slightly more than 1 point higher, on average, holding all else equal. Similar findings did not emerge for the obesity model.

With respect to usual participation in the SBP, the association between participating 3 days per week in comparison to 0 days per week was found to be statistically significant and positive in both the BMI model and the obesity model (Table 5). However, none of the other associations were statistically significant. In addition, the coefficients across participation levels did not exhibit a clear trend in any model, which reinforces the results from the binary model which indicate no clear association between SBP participation and student weight status.

## 4. Discussion

This paper sought to estimate the relationship between usual participation in the school meal programs and student weight status, in order to assess how these relationships may have changed since the implementation of the updated meal patterns. Studies have shown that the nutritional quality of school meals has improved as a result of the updated meal standards, particularly with regard to the provision of whole grains and greens and beans [7] and reductions in sodium content [19,20]. Importantly, the meals that participants consumed were significantly more nutritious than the meals that nonparticipants consumed from home or somewhere else, which suggests the possibility that participation in the NSLP may reduce the likelihood of childhood obesity. However, this analysis found no association between usual participation in the NSLP, defined as 3 or more days per week, and student weight status. The models controlled for a wide array of student characteristics along with school-level fixed effects, making it less likely that this association was the result of selection bias influencing who participates in the NSLP, although it is still possible that unobserved differences between participants and nonparticipants could have led to bias in these estimates.

Compared to usually participating in the NSLP 0 days per week, all levels of usual participation were positively associated with BMI; however, not all were significant. In addition, these associations did not strictly increase with a higher number of days of participation, indicating a nonlinear relationship between participation and weight status. For example, holding everything else equal, students who usually participate in the NSLP twice a week were expected to have BMIs which were approximately 2 points higher, on average, than those who participate 0 days per week, whereas students who usually participate in the NSLP 3 days per week were expected to have BMIs which were about 0.4 points higher, on average, compared to those who participate 0 days per week. Similarly, results from the bivariate analysis show that roughly equal proportions of students who usually consume two, three, four, or five school lunches each week were affected by overweight or obesity. If student weight status were associated with NSLP participation, one would expect consuming more meals per week to consistently lead to higher rates of overweight and obesity. This was not the case. Therefore, combined with the finding that there was no statistically significant difference in weight status for students who usually participated in the NSLP 3 or more days per week compared with those who participated less often, these findings suggest that school lunches do not lead to higher BMIs.

Although it was outside the scope of the current analysis to establish whether meal participation caused student weight gain, recent research spanning school years 1998–1999 through 2006–2007 explored the causal impact of NSLP and SBP participation on BMI among low-income elementary- and middle-school-aged children using a difference-in-difference model [10]. In particular, Capogrossi and You (2017) found mixed evidence on the effect of participation in the NSLP and SBP on student BMI among children from households at or below 185% of poverty. Although they found that long-term participation in both the SBP and the NSLP (participation from 1st through 8th grade) significantly increased the probability of being overweight, they found no significant association between BMI and shorter-term participation in the NSLP (participation from 1st through 5th grade), nor did they find any statistically significant association between BMI and switching from not participating to participating in the NSLP between 5th and 8th grade. Additional research is needed to better understand the causal mechanism between meal participation and student weight status across all students.

Similar to the NSLP findings, the current analysis found no statistically significant associations between usual SBP participation and weight status. Across all model specifications, the magnitude of the association between usual SBP participation and students’ weight status was small and not statistically significant. Because the SNMCS did not collect information related to whether students usually consume breakfast, it was not possible to explore how SBP participation, separate from usual breakfast skipping, may be associated with student weight status. Had we been able to control for this behavior, different results may have emerged.

To replicate the findings of Gleason and Dodd’s original analysis, we also examined the association between weight status and participation using a continuous measure of participation (see Appendix A Table A3). However, there was no clear linear association between weight status across the days of participation, suggesting that treating participation as a continuous measure is not ideal. Nevertheless, a significant relationship between NSLP participation, but not SBP participation, and weight status was observed. Specifically, for each additional day of usual NSLP participation, a 0.2-point increase in students’ BMI is expected, which is equivalent to a 1-point higher BMI for a student who usually participates every day in the NSLP compared to a student who never participates. Importantly, the results presented in this paper differed from those presented by Gleason and Dodd (2009) [6], who did not observe any statistically significant association between NSLP participation and student weight status. Instead, their research found the association between BMI and SBP participation was statistically significant and negative, but this relationship was primarily driven by non-Hispanic, white children. Although it may be tempting to believe that changes in the nutritional standards of school meals, in response to the HHFKA, may be responsible for these differences, it is likely that changes in the composition of who participates in the school meal programs contribute to these differences.

As research has shown, there has been a persistent downward trend in NSLP participation among students who do not qualify for free and reduced-price meals since school year 2007–2008, which predates the passage of the HHFKA [9]. In particular, the Food Research and Action Center identifies two likely reasons for this trend: (1) the Great Recession of 2007, which led to more children qualifying for free- and reduced-priced meals, who had previously received paid meals, and (2) increased pricing of paid meals between school year 2007–2008 and school year 2013–2014, which discouraged paid participants from continuing to participate. Indeed, comparisons of student characteristics among usual NSLP participants in Gleason and Dodd’s dataset (School Nutrition and Dietary Assessment III) and SNMCS reveal statistically significant and substantial declines among students from households with incomes above 185% of the poverty level as well as among non-Hispanic, white students [5].

Given that the characteristics of students who participated in the school meal programs have meaningfully changed over time, a key limitation in this research is the inability to disentangle what may be driving the changes in the associations between participation and weight status in the current analysis from those observed in Gleason and Dodd’s previous work. Although the current analysis controls for a wide array of student characteristics and school fixed effects, it is possible that omitted student or household characteristics might impact students’ decisions to participate in the meal program as well as their weight status. Furthermore, given that the passage of the HHFKA coincided with changing sociodemographic characteristics of student participants, it is not clear what role, if any, changes in the nutritional quality of meals had on student weight status independent of the changes in the characteristics of students who participated in the school meal programs. Future research should seek to distill the effects of the updated nutrition standards independent of the effects on participant composition.

There is great diversity in school food environments available to students; therefore, it may also be worthwhile to explore how differences in school-level policies, such as adherence to the revised nutritional standards or nutrition education and promotion, are associated with student weight status. However, while the school meal programs contribute substantially to participants’ diets, many factors impact student weight status. As a result, improvements in the nutritional quality of school meals alone without changes in meal consumption outside of school or activity level may not be sufficient to impact weight status. In addition, increased nutritional quality of students’ diets in SNMCS were measured using the HEI, which is not a measure of energy intake. Therefore, it is not unexpected that a clear relationship between improved diet quality and students’ weight status did not emerge, particularly in the short-term. More research is needed to understand the role that school meals may have in reducing childhood obesity, given the complexity in the relationship between energy intakes and expenditures and weight status.

## 5. Conclusions

Overall, this paper provides a much-needed re-examination of the relationship between student weight status and school meal participation since the passage of the HHFKA. Using a nationally representative sample of students and a rich set of student characteristics and school fixed effects, this research shows no clear association between usual participation in the school meal programs and students’ weight status, which contradicts the findings from previous research conducted before the HHFKA. Notably, the quality of school meals and the composition of the students who consume them have significantly changed over time, making it difficult to discern which factors, or combination of factors, have led to these differences. Therefore, future research should seek to build upon these initial findings to fully elucidate the causal mechanism between participation in school meal programs and student weight status.

## Figures and Tables

**Table 1:** Percentage of school-age children by participation status and meal type.

	NSLP (*N* = 1963)	SBP (*N* = 1963)
Number of Days	% (Standard Error)	
0 days ^a^	27.8 (±2.3)	61.2 (±2.9)
1 day	8.5 (±1.0)	7.0 (±1.0)
2 days	5.8 (±0.9)	6.3 (±0.7)
3 days	8.0 (±0.7)	7.1 (±0.9)
4 days	6.7 (±0.9)	5.1 (±0.8)
5 days	43.3 (±2.7)	13.4 (±1.5)

^a^ Includes students who attend schools that do not offer the School Breakfast Program (SBP).

**Table 2:** Characteristics of students, by National School Lunch Program (NSLP) and SBP usual participation status.

	NSLP		SBP	
	Usual Non-Participant (*N* = 742)	Usual Participant (*N* = 1221)	Usual Non-Participant (*N* = 1467)	Usual Participant (*N* = 496)
	% (Standard Error)			
Female	54.2 (±2.6)	45.6 *** (±2.0)	51.1 (±1.8)	43.4 (±3.0)
Race and ethnicity ^a,b^				
Hispanic	15.6 (±2.0)	29.9 (±3.5)	20.0 (±2.4)	33.9 (±4.8)
Non-Hispanic, white	67.8 (±2.8)	48.2 (±4.1)	62.6 (±2.9)	36.9 (±5.2)
Non-Hispanic, black	7.1 (±1.5)	15.1 (±3.1)	8.4 (±1.7)	22.6 (±5.2)
Other	9.5 (±1.4)	7.6 (±0.9)	9.0 (±1.1)	6.7 (±1.4)
Age	13.0 (±2.6)	11.6 *** (±1.8)	12.7 (±2.0)	10.6 *** (±2.6)
Food security ^a,b^				
Food secure	91.6 (±1.5)	77.0 (±1.7)	87.0 (±1.5)	70.8 (±2.4)
Low food secure	6.2 (±1.3)	17.4 (±1.4)	9.8 (±1.2)	22.2 (±2.3)
Very low food secure	2.2 (±0.5)	5.6 (±0.9)	3.2 (±0.6)	7.1 (±1.5)
Income-to-poverty level ^a,b^				
More than 185% ofpoverty threshold	82.0 (±2.5)	42.7 (±2.9)	68.9 (±2.4)	28.7 (±3.1)
More than 130% to 185%of poverty threshold	4.7 (±1.1)	11.6 (±1.4)	7.8 (±0.8)	11.5 (±2.1)
Less than or equal to130% of poverty threshold	13.3 (±1.9)	45.6 (±2.6)	23.2 (±2.0)	59.8 (±3.4)

**Note:** Usual NSLP participation is defined as participating in the NSLP for 3 or more days per week. Usual SBP participation is defined as participation in the SBP for 3 or more days per week. Overweight or obese is defined as at or above 85th percentile of BMI-for-age reference. *** *p* < 0.01 ^a^ Comparisons between NSLP usual participants and nonparticipants are statistically significant (*p* < 0.01). ^b^ Comparisons between SBP usual participants and nonparticipants is statistically significant (*p* < 0.01). Pearson chi-square tests were used to compare differences between categorical variables and two-sided t-tests were used to compare differences between continuous variables.

**Table 3:** Percentage of school-age children classified as overweight or obese, by school type.

		Elementary (*N* = 675)	Middle (*N* = 646)	High (*N* = 642)	All (*N* = 1963)
	*N*	% (Standard Error)			
Overall	1963	37.4 (±1.7)	34.9 (±2.7)	32.4 (±2.7)	35.2 (±1.5)
Gender					
Male	1031	36.6 (±3.4)	39.1 (±3.9)	35.0 (±3.8)	36.6 (±2.3)
Female	932	38.2 (±3.1)	30.4 (±3.0)	29.7 (±3.9)	33.7 (±2.1)
Race and ethnicity					
Hispanic	499	46.5 (±3.4)	35.7 (±5.7)	31.3 (±5.9)	41.1 (±2.5)
Non-Hispanic, white	1051	30.3 (±3.3)	34.0 (±3.5)	31.7 (±3.6)	31.7 (±2.2)
Non-Hispanic, black	233	44.3 (±5.9)	43.0 (±7.4)	35.1 (±6.7)	41.0 (±3.1)
Other	180	33.0 (±7.9)	31.0 (±7.8)	36..4 (±9.1)	33.7 (±5.0)
Usual NSLP participation					
0 days	465	22.8 (±5.4)	25.0 (±3.7)	26.2 (±4.3)	25.0 (±3.1)
1 day	154	30.7 (±7.1)	26.1 (±6.7)	26.7 (±8.8)	28.3 (±6.9)
2 days	115	46.0 (±8.8)	41.5 (±9.6)	43.5 (±10.0)	44.0 (±8.1)
3 days	179	33.5 (±8.4)	52.0 (±9.5)	39.4 (±6.7)	38.9 (±5.4)
4 days	151	42.3 (±7.7)	40.9 (±9.9)	36.2 (±7.5)	40.2 (±4.8)
5 days	899	42.3 (±2.6)	38.6 (±3.5)	37.9 (±3.9)	40.4 (±1.8)
Usual SBP participation					
0 days ^a^	1174	35.0 (±3.0)	31.3 (±3.0)	32.3 (±3.3)	32.9 (±2.0)
1 day	135	34.1 (±6.9)	47.8 (±12.2)	11.8 (±5.2)	31.1 (±6.7)
2 days	130	34.7 (±8.9)	33.5 (±9.6)	30.6 (±8.2)	32.9 (±7.4)
3 days	150	51.8 (±8.1)	54.6 (±11.1)	43.3 (±8.8)	50.4 (±7.6)
4 days	113	41.9 (±8.5)	34.5 (±11.8)	44.4 (±15.6)	41.4 (±9.2)
5 days	261	36.5 (±4.0)	44.1 (±10.9)	37.1 (±7.6)	37.5 (±3.6)

**Note:** Overweight or obese is defined as at or above 85th percentile of BMI-for-age reference. ^a^ Includes students who attend schools that do not offer the SBP.

**Table 4:** Estimated association between usually participating in the SBP and the NSLP 3 days per week on BMI.

	Dependent Variable		
	BMI	Overweight or Obese	Obese
	Coefficient (Standard Error)		
Usual NSLP participation	0.37 (±0.28)	0.31 (±0.18)	−0.10 (±0.24)
Usual SBP participation	0.58 (±0.38)	0.21 (±0.24)	0.29 (±0.30)
Controls ^a^	Included	Included	Included
R^2^	0.44	—	—
Observations	1963	1834	1478

**Note:** Usual NSLP participation is defined as participating in the NSLP for 3 or more days per week. Usual SBP participation is defined as participation in the SBP for 3 or more days per week. Overweight or obese is defined as at or above 85th percentile of BMI-for-age reference. Coefficients for the overweight or obese and the obesity models are in logged odds. ^a^ Includes all covariates listed in Table 1 in addition to school-level fixed effects.

**Table 5:** Estimated association between SBP and NSLP participation on BMI, categorical.

	Dependent Variable		
	BMI	Overweight or Obese	Obese
	Coefficient (Standard Error)		
Usual NSLP participation			
0 days (reference category)			
1 day	0.63 (±0.54)	0.67 (±0.39)	0.21 (±0.45)
2 days	1.68 ** (±0.65)	1.24 *** (±0.33)	1.34 *** (±0.49)
3 days	0.41 (±0.51)	0.61 (±0.36)	0.06 (±0.41)
4 days	1.17 ** (±0.55)	1.00 *** (±0.37)	0.23 (±0.52)
5 days	1.07 *** (±0.36)	0.789 *** (±0.21)	0.50 (±0.29)
Usual SBP participation			
0 days (reference category)			
1 day	−0.14 (±0.43)	−0.23 (±0.33)	−0.18 (±0.45)
2 days	0.57 (±0.64)	−0.29 (±0.41)	0.63 (±0.40)
3 days	1.85 *** (±0.59)	0.66 (±0.37)	1.12 ** (±0.43)
4 days	0.62 (±0.75)	0.07 (±0.47)	0.37 (±0.57)
5 days	−0.09 (±0.45)	−0.04 (±0.25)	−0.19 (±0.36)
Controls ^a^	Included	Included	Included
R^2^	0.45	—	—
Observations	1963	1834	1478

**Note:** Overweight or obese is defined as at or above 85th percentile of BMI-for-age reference. Coefficients for the overweight or obese and the obesity models are in logged odds. ^a^ Includes all covariates listed in Table 1 in addition to school-level fixed effects. ** *p* < 0.05 *** *p* < 0.01.

## Data Availability

The data presented in this study are restricted-use and come from a large, nationally representative study of the school meal programs that operate in the United States. Requests for access to the public use version of these data should be submitted via electronic mail to: FNSStudies@usda.gov.

## Appendix A

**Table A1:** Proportion of dietary intake-day participants by usual days of participation in the NSLP and SBP.

	*N*	NSLP Dietary Intake-Day Participant	*N*	SBP Dietary Intake-Day Participant
Days of Usual Participation		% (Standard Error)		
0 days ^a^	465	7.0 (±1.6)	1174	2.5 (±0.6)
1 day	154	29.7 (±5.3)	135	20.7 (±4.2)
2 days	115	44.6 (±6.2)	130	34.3 (±4.9)
3 days	179	64.1 (±4.9)	150	43.0 (±5.8)
4 days	151	79.1 (±3.9)	113	59.7 (±6.6)
5 days	899	87.7 (±1.8)	261	67.9 (±4.8)

^a^ Includes students who attend schools that do not offer the SBP.

**Table A2:** Student and household characteristics included as model controls.

Student Demographics		
Variable	Type	Categories/Range ^a^
Age	Continuous	[6,19]
Gender	Binary	Female Not female
Interaction between age and gender	Interaction	n.a.
Race and ethnicity	Categorical	Hispanic White, non-Hispanic Black, non-Hispanic Other
**Student physical activity and general health status**		
Parent-reported measure of student’s health	Categorical	Health is excellent Health is very good Health is good, fine, or poor
Parent-reported measure of student activity level	Categorical	Less active than others same age As active as others same age More or much more active than others same age
Physical education classes per week	Categorical	0 days per week 1 day per week 2 days per week 3 days per week 4 days per week 5 days per week
No. of days per week spent watching at least 60 min of television	Categorical	0 days per week 1 day per week 2 days per week 3 days per week 4 days per week 5 days per week
No. of days per week spent playing computer games for at least 60 min	Categorical	0 days per week 1 day per week 2 days per week 3 days per week 4 days per week 5 days per week
**Student’s usual eating habits**		
Parent-reported measure of whether student is a picky eater	Categorical	Very picky eater Somewhat picky eater Not a picky eater
Parent-reported measure of amount student eats	Categorical	Larger amount than others the same age Same amount as others the same age Smaller amount as others the same age
Student’s family usually has skim or low-fat milk	Binary	Yes No
Student’s family regularly serves butter/margarine/sour cream when they have potatoes	Binary	Yes No
Student has food allergies	Binary	Yes No
Student has dieted in last 30 days	Binary	Yes No
**Household characteristics**		
Receipt of public assistance	Binary	Yes No
Income-to-poverty level	Categorical	More than 185% of poverty threshold More than 130% to 185% of poverty threshold Less than or equal to 130% of poverty threshold
Food security	Categorical	Food secure Low food secure Very low food secure
Primary language spoken at home	Categorical	English Other

^a^ Categories are provided for binary and categorical variables, and the minimum and maximum values are provided for continuous variables.

**Table A3:** Estimated association between SBP and NSLP participation on BMI, continuous.

	Dependent Variable		
	BMI	Overweight or Obese	Obese
	Coefficient (Standard Error)		
Usual NSLP participation	0.20 *** (±0.07)	0.13 *** (±0.04)	0.08 (±0.05)
Usual SBP participation	0.08 (±0.08)	0.02 (±0.05)	0.03 (±0.07)
Controls ^a^	Included	Included	Included
R^2^	0.44	—	—
Observations	1963	1834	1478

**Note:** Usual NSLP and SBP participation are defined in terms of the number of days of usual participation, ranging from 0 to 5 days. Overweight or obese is defined as at or above 85th percentile of BMI-for-age. ^a^ Includes all covariates listed in Table 1 in addition to school-level fixed effects. *** *p* < 0.01.
